# Balloon pulmonary angioplasty – efficient therapy of chronic thromboembolic pulmonary hypertension in the patient with advanced sarcoidosis – a case report

**DOI:** 10.1186/s12890-018-0695-4

**Published:** 2018-08-16

**Authors:** Andrzej Labyk, Dominik Wretowski, Sabina Zybińska-Oksiutowicz, Aleksandra Furdyna, Katarzyna Ciesielska, Dorota Piotrowska-Kownacka, Olga Dzikowska –Diduch, Barbara Lichodziejewska, Andrzej Biederman, Piotr Pruszczyk, Marek Roik

**Affiliations:** 1 Center for Diagnostics and Treatment of Venous Thromboembolism, Department of Internal Medicine and Cardiology, Warsaw Medical University, Infant Jesus Hospital, Lindleya Street 4, 02-005 Warsaw, Poland; 2 Department of Radiology, Infant Jesus Hospital, Lindleya Street 4, 02-005 Warsaw, Poland; 3 Cardiac Surgery Department Medicover Hospital, Rzeczypospolitej 5 Avenue, 02-972 Warsaw, Poland

**Keywords:** Sarcoidosis, Chronic thromboembolic pulmonary hypertension, Balloon pulmonary angioplasty

## Abstract

**Background:**

Approximately a quarter of patients with advanced sarcoidosis develop pulmonary hypertension (PH), which affects their prognosis. We report unusual case of confirmed chronic thromboembolic pulmonary hypertension (CTEPH) in a patient with stage IV sarcoidosis successfully treated with balloon pulmonary angioplasty (BPA).

**Case presentation:**

A 65 years old male with a history of colitis ulcerosa, and pulmonary sarcoidosis diagnosed in 10 years before, on long term oral steroids, with a history of deep vein thrombosis and acute pulmonary embolism chronically anticoagulated was referred to our center due to severe dyspnea. On admission he presented WHO functional class IV, mean pulmonary artery pressure (mPAP) in right heart catheterization (RHC) was elevated to 54 mmHg. Diagnosis of CTEPH was definitely confirmed with typical V/Q scan, and with selective pulmonary angiography (PAG) completes by intravascular imagining (intravascular ultrasound, optical coherent tomography). The patient was deemed inoperable by CTEPH team and two sessions of BPA with multimodal approach resulted in significant clinical and haemodynamical improvement to WHO class II and mPAP decrease to 27 mmHg.

**Conclusions:**

Balloon pulmonary angioplasty, rapidly developing method of treatment of inoperable CTEPH patients, is also extremely useful therapeutic tool in complex PH patients.

## Background

Approximately 26% of patients with advanced pulmonary sarcoidosis may develop pulmonary hypertension (PH), which is related with poor prognosis [1, 2]. According to current guidelines, patients with suspected PH should undergo detailed diagnostic workup and differential diagnosis among others should include chronic lung diseases leading to hypoxia and chronic thromboembolic pulmonary hypertension (CTEPH) [3]. We report an unusual challenging case of CTEPH in a patient with stage IV sarcoidosis.

## Case presentation

**Fig. 1:**
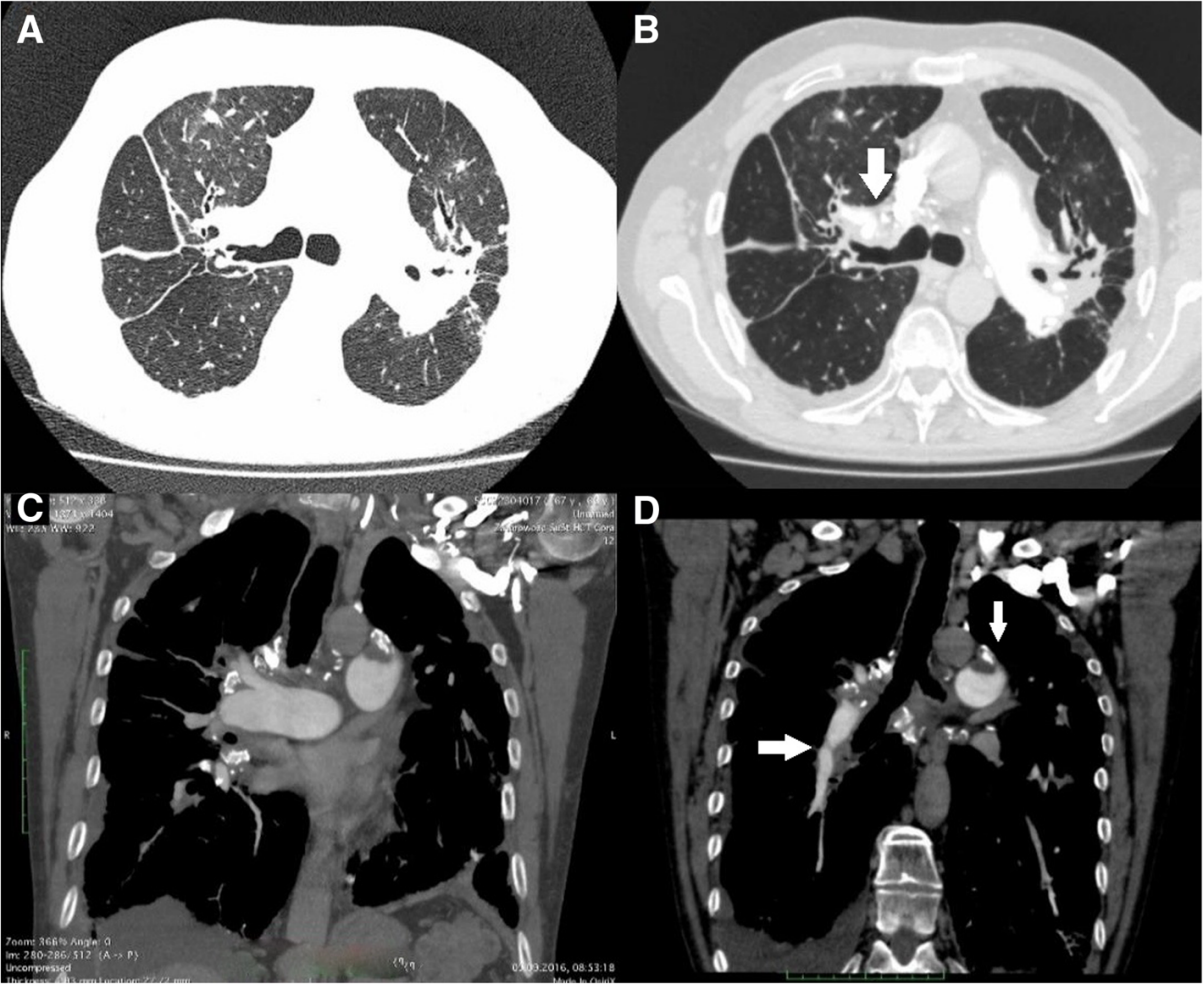
**a, b** – interstitial lung fibrosis and calcified lymph nodes. **c, d** – organized thrombi in left and right pulmonary arteries

**Fig. 2:**
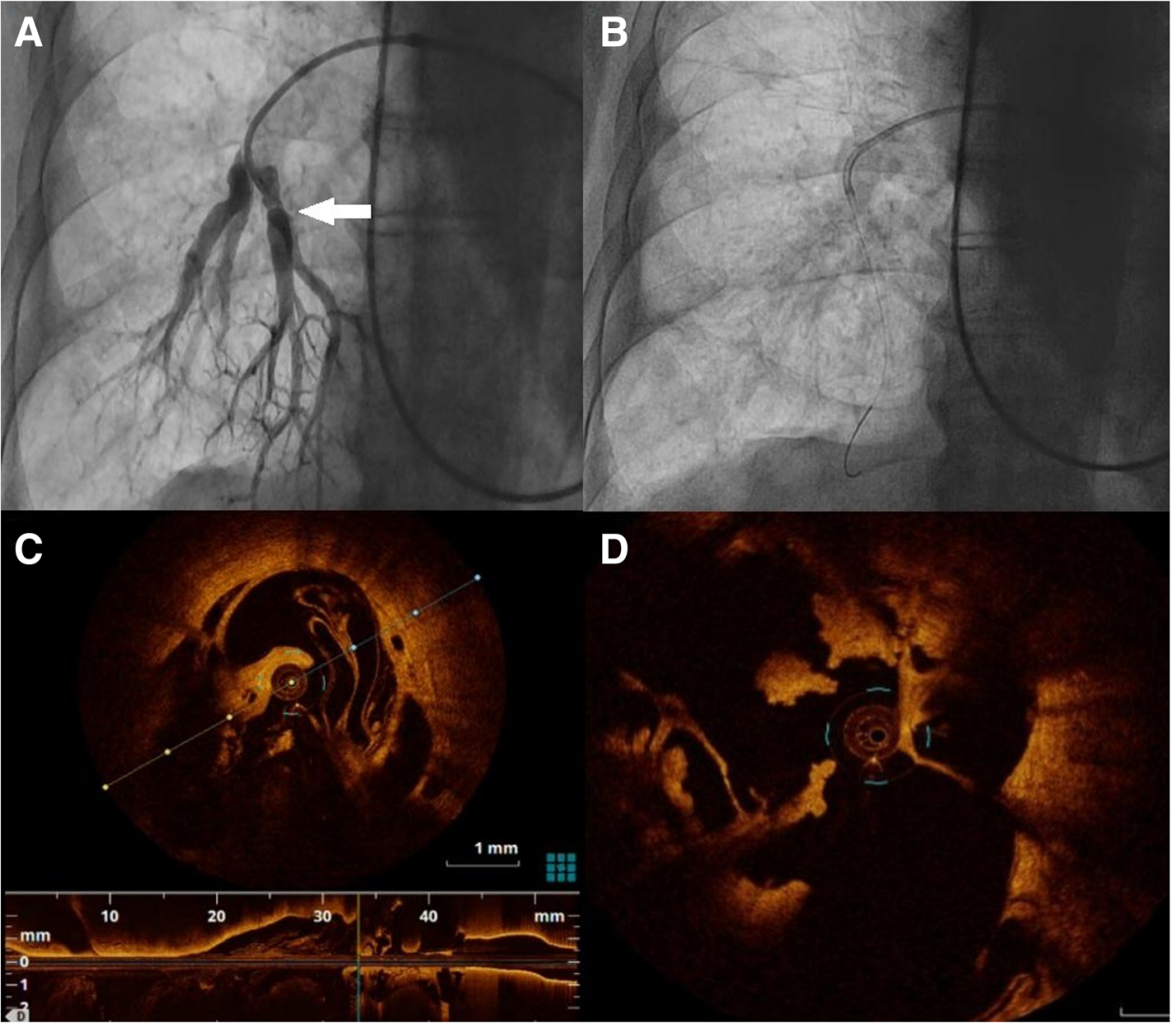
**a** – initial selective right pulmonary artery angiography with intravascular web marked with arrow. **b, c** – intravascular web/organized thrombus in optical coherent tomography (OCT). **d** – guide wire with pressure catheter to assess pulmonary pressure ratio (PPR)

A 65 years old male with history of colitis ulcerosa, and advanced sarcoidosis diagnosed 10 years before, on oral steroids was admitted to our department due to exertional dyspnea and right ventricular (RV) heart failure progressing since last 12 months to functional class IV. Two years earlier, he experienced the first severe decompensation of right heart function. At that time PH was diagnosed on echocardiography. RV to left ventricle (LV) ratio exceeded one (RV:LV - 39/32 mm); peak systolic tricuspid regurgitation gradient (TRPG) was 75 mmHg, and decreased tricuspid annular plane systolic excursion (TAPSE) 15 mm indicated significant pressure overload and RV systolic dysfunction. Chest computed tomography (CT) revealed sarcoidosis progression, however no pulmonary thromboemboli were detected. After typical heart failure treatment with diuretics, ACE inhibitors, and steroid dose increase the patient improved and he was discharged home in a good clinical condition in WHO functional class II, with the diagnosis of PH due to sarcoidosis stage IV. One year later, due to acute dyspnea, worsening of RV function and unilateral leg swelling he underwent another chest CT, which this time showed fresh thrombi in the left segmental upper lobe pulmonary artery. Moreover, acute deep vein thrombosis was detected with lower limb compression ultrasound. Long term oral anticoagulation with rivaroxaban was started. Two years later the patient was referred to our center due to progressive functional deterioration. On admission he was in WHO functional class IV, saturation on room air was 85%, blood pressure 120/80 mmHg, heart rate 90 beats per minute. Mild peripheral edema was present. Distance covered in 6 min walk test (6MWT) was 100 m with desaturation to 77%. Plasma natriuretic peptide (NT-pro-BNP) concentration was elevated to 6239 pg/ml (normal range < 125 pg/ml). Echocardiography showed severe RV pressure overload with TRPG 95 mmHg, dilatation of both right atrium and ventricle. Preserved morphology and function of LV was observed. Chest CT scan showed signs of advanced interstitial lung fibrosis (Fig. 1a) and calcified mediastinal lymph nodes (Fig. 1b). However, organized thrombi in both pulmonary were also detected (Fig. 1c and d). At that time multifactorial etiology of PH was considered: sarcoidosis with secondary PH and local in situ thrombosis, or CTEPH in a patient with stage IV sarcoidosis, and with deep vein thrombosis in the past. Lung perfusion scan with SPECT/CT showed bilateral perfusion defects which strongly suggested thromboembolic component of PH. Right heart catheterization (RHC) followed by selective pulmonary angiography (PAG) showed mean pulmonary artery pressure (mPAP) of 54 mmHg. Pulmonary artery wedge pressure (PAWP) was 6 mmHg and pulmonary vascular resistance (PVR) was 13,5 Wood Units. Selective PAG confirmed chronic thromboembolic lesions suggestive of CTEPH – left upper lobe artery occlusion, intravascular webs in right pulmonary artery (Fig. 2a). After experienced cardiac surgeon consultation, the patient was deemed inoperable due to advanced, sarcoidosis - related lung changes and propable complications regarding the use of extracorporeal circulation. He was finaly qualified for balloon pulmonary angioplasty (BPA).

**Fig. 3:**
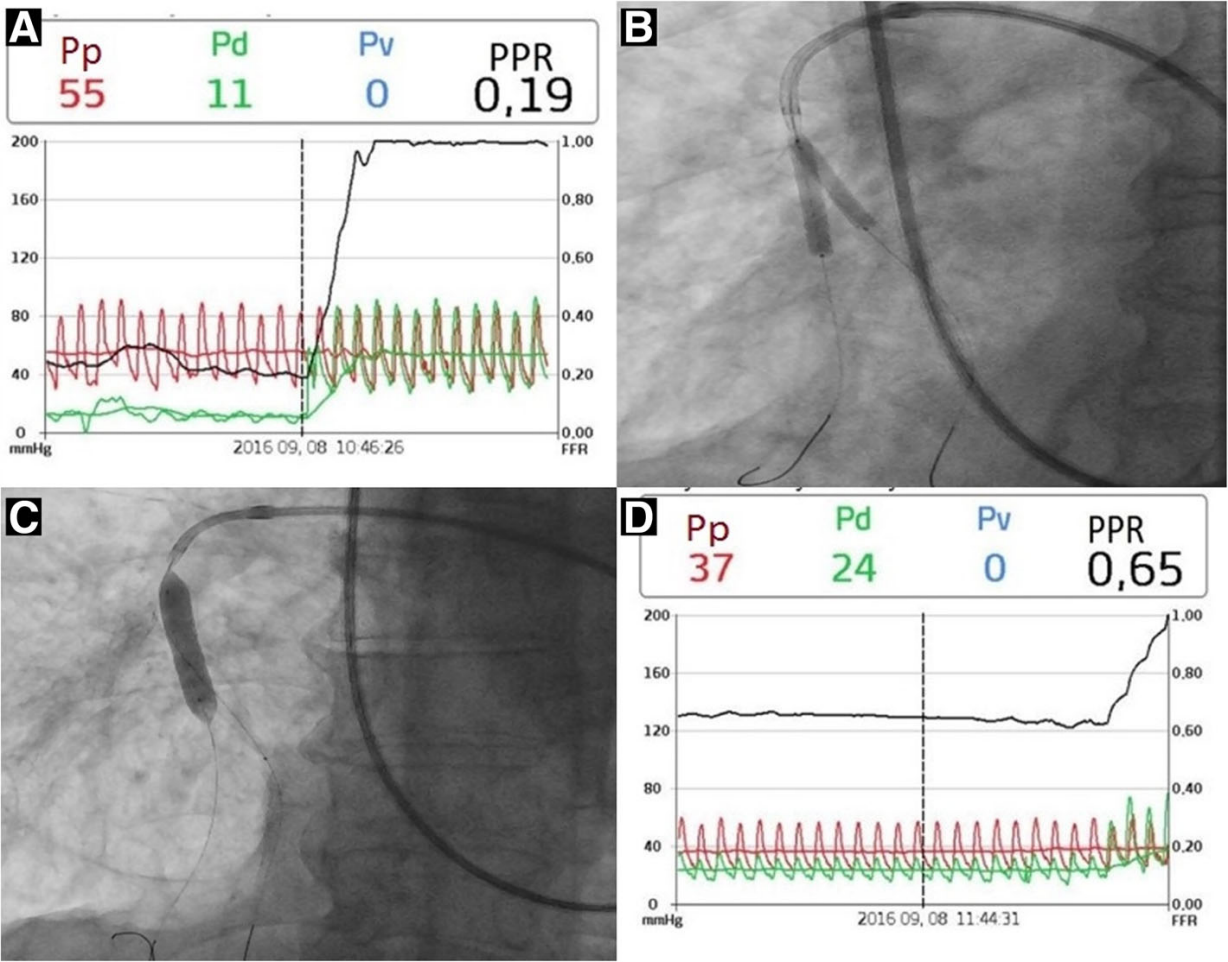
**a** – PPR assessment before balloon pulmonary angioplasty, green curve nearly flat at first with rise after thrombus crossing. **b** – “kissing balloons” technique. **c** – proximal optimalization with large balloon catheter. **d** – final PPR assessment, green curve showes restored pressure on safe level, preventing reperfusion pulmonary injury

BPA procedures were performed according the previously published protocol [4]. Importantly, in order to confirm intraluminal localization of thromboembolic lesions intravascular imaging with optical coherence tomography (OCT) and intravascular ultrasound (IVUS) were performed. It allowed to definitely confirm organized thrombi (Fig. 2a – white arrow, 2C, 2D). With the use of pressure catheter we assessed hemodynamic significance of intrapulmonary lesions (Fig. 2b). Pulmonary pressure ratio (PPR, the ratio of the pressure distal to the lesion (Pd) divided by the pressure proximal to the lesion (Pp)) was assessed to optimize the BPA procedure and to minimize potential complications such as reperfusion pulmonary injury (RPI). PPR in arteries A9 and A10 was 0.19 and 0.22 – which suggested functional occlusion of both arteries. After simultaneous inflation of balloon catheters - “kissing balloon” technique (4.0x20mm and 4.0x27mm respectively), followed by proximal optimization with 7.0x30mm balloon catheter resulted in PPR 0.63 in A9 and 0.65 in A10 (Fig. 3, part A and D – green curve marking distal pressure). There were no periprocedural complications. Mean PAP after the first BPA procedure decreased from 52 mmHG to 40 mmHg. The second BPA session was performed 3 weeks later (left pulmonary artery), also without complications. Both procedures (3 segmental lesions in total) resulted in further hemodynamic and functional improvement, with mPAP drop to 27 mmHg at 12 months follow – up. Patient was still in functional class II WHO, echocardiography and 6MWT showed further improvement in clinical condition and RV function (Table 1).

**Table 1:** Clinical and hemodynamical assessment before and after 2 BPA sessions

	Before treatment	At 12 months follow-up
WHO functional class	IV	II
NT-pro-BNP level (pg/ml)	6239	281
6MWT distance (m)	100	405
TRPG (mmHg)	95	31
RVSP (mmHg)	102	53
TAPSE (mm)	14	20
AcT (ms)	63	100
mPAP (mmHg)	54	27
CI (l/min/m^2^)	2.08	3.34
RAP (mmHg) systolic/diastolic/mean	14/10/9	5/3/1
PVR (Wood Units)	13.5	3.7

*NT-pro-BNP* natriuretic peptide concentration, *6MWT* 6 min walk test, *TRPG* tricuspid regurgitation peak gradient, *RVSP* right ventricle systolic pressure, *TAPSE* tricuspid annular plane excursion, *AcT* acceleration time, *mPAP* mean pulmonary artery pressure, *CI* cardiac index, *RAP* right atrial pressure, *PVR* pulmonary vascular resistance, *BPA* balloon pulmonary angioplasty

## Discussion and conclusions

Sarcoidosis related PH as is predominantly caused severe lung fibrosis or by compression of pulmonary arteries by mediastinal fibrosis and lymphadenopathy [1, 2, 5]. Pulmonary artery stenting was reported to be efficient in the treatment of pulmonary arteries compression in advanced sarcoidosis [5], while pharmacology with sildenafil or other PH specific dugs are of very limited short term efficacy [6, 7].

Presented case is probably one of the first available reports with initially suspected sarcoidosis related PH, which eventually was verified by pulmonary angiography and multimodal intravascular assessment as CTEPH. There are no data on optimal treatment in such challenging patients. After being deemed inoperable due to advanced sarcoidosis resulting in severe lung alterations, the patient was qualified to BPA procedure. Percutaneous catheter based method, is a rapidly developing therapeutic method for CTEPH patients. There is a growing evidence that in expert centers it is safe and effective, allowing normalization or near normalization of mPAP in majority of treated cases [4, 8–13]. In our institution we have started BPA program in inoperable CTEPH in 2014. BPA procedures are performed according to the previously described protocol [4] with the use of pressure catheter, and in selected cases with OCT/IVUS intravascular imaging. Our data indicate that this technique is safe and effective also in CTEPH high risk patients with severe comorbidities [14]. We are convinced that multimodal approach not only allowed to confirm CTEPH definitely in the patient with advanced sarcoidosis, but it also allowed to treat him and successfully and safely. We performed angioplasties of selected thromboembolic lesions, which were proved to be hemodynamically significant in multimodal assessment. Such fast improvement was also observed in other CTEPH patients treated with BPA. Based on our experience, it is related with initial treatment of the lesions in segmental artreries of lower lobes. In presented case, specific drug therapy (riociguat) was not avaliable at that time. Patient was on sildenafi (25 mg t.i.d.) and initial mPAP decreased only by 2 mmHg (54 mmHg in initial RHC versus 52 mmHg before first BPA session). Thus, only two BPA sessions resulted in mPAP reduction and significant clinical improvement. We think that BPA became a real therapeutic option in CTEPH patients, who are not candidates for surgery.

## Abbreviations

6MWT: 6 min walk test
AcT: Acceleration time
BPA: Balloon pulmonary angioplasty
CI: Cardiac index
CT: Computed tomography
CTEPH: Chronic thromboembolic pulmonary hypertension
IVUS: Intravascular ultrasound
LV: Left ventricle
mPAP: Mean pulmonary artery pressure
NT-pro-BNP: Natriuretic peptide concentration
OCT: Optical coherent tomography
PAG: Pulmonary angiography
PAWP: Pulmonary artery wedge pressure
PH: Pulmonary hypertension
PPR: Pulmonary pressure ratio
PVR: Pulmonary vascular resistance
RAP: Right atral pressure
RHC: Right heart catheterization
RPI: Reperfusion pulmonary injury
RV: Right ventricle
RVSP: Right ventricle systolic pressure
SPECT: Single-photon emission computed tomography
TAPSE: Tricuspid annular plane excursion
TRPG: Tricuspid regurgitation peak gradient
